# Cord blood and amniotic membrane extract eye drop preparations display immune-suppressive and regenerative properties

**DOI:** 10.1038/s41598-021-93150-7

**Published:** 2021-07-02

**Authors:** Dinara Samarkanova, Steven Cox, Diana Hernandez, Luciano Rodriguez, Maria Luisa Pérez, Alejandro Madrigal, Anna Vilarrodona, Sergio Querol, Ricardo P. Casaroli-Marano

**Affiliations:** 1 Banc de Sang i Teixits, Passeig Taulat 116, 08005 Barcelona, Spain; 2 Transfusional Medicine Research Group, Vall d’Hebron Research Institute (VHIR), Barcelona, Spain; 3 Department of Medicine, Universidad Autónoma de Barcelona (UAB), Barcelona, Spain; 4 Anthony Nolan Research Institute, Royal Free Hospital, London, UK; 5 UCL Cancer Institute, Royal Free Campus, London, UK; 6 Barcelona Tissue Bank, Banc de Sang i Teixits, Barcelona, Spain; 7 Regenerative Medicine and Tissue Bioengineering Research Group (SGR-1113), Biomedical Research Institute (IIB-Sant Pau), Barcelona, Spain; 8 Department of Surgery, School of Medicine and Hospital Clinic de Barcelona, University of Barcelona, Barcelona, Spain

**Keywords:** Immunology, Medical research

## Abstract

Diseases and injuries that compromise the ocular surface cause considerable patient distress and have long term consequences for their quality of life. Treatment modalities that can address the delicate balance of tissue regeneration, inflammation and maintenance of corneal transparency are therefore needed. We have recently formulated two novel eye drops from placental tissues: cord blood platelet lysate (CBED) and amniotic membrane extract eye drops (AMED), which can be used to treat severe ocular disorders. Here we characterise these two preparations by measuring: (a) growth factors (GF) and cytokines composition, (b) promotion of human corneal epithelial cell (HCEC) growth and (c) effects on immune cells in a lymphocyte culture assay. Finally, their bioavailability was assayed in an ex vivo porcine corneal model. We show that both preparations contain GF and cytokines that were able to promote the in vitro growth of HCEC and support repair in an in vitro scratch test. When assessed in a lymphocyte culture, both favoured immune suppression reducing the cellular expression of NKG2D and CD107a as well as the production of interferon gamma (IFN-γ) in natural killer, NKT and T cells. Regarding bioavailability, CBED active molecules were found mainly in the pre-corneal fraction with some penetration into the corneal fraction, in an ex vivo model. In summary, both placental-derived allogeneic preparations, CBED and AMED, display regenerative and immunomodulatory capabilities. These results will help define mechanisms of action and the best indications and doses of each product for use in a particular patient and support the development of off-the-shelf therapies for ocular surface pathologies in which wound healing defects and inflammatory events are contributing factors.

## Introduction

The integrity of the ocular surface is crucial for the maintenance of corneal transparency, physiology, and the preservation of vision. Thus, ocular surface disorders, which may vary from mild dry eye disease to neurotrophic ulcers and corneal surgery, lead to dysfunctions that cause significant symptoms for the patient, including compromised vision^1–4^. It has been observed that components of human tissues, such as blood and amniotic membrane, are enriched with molecules similar to those found in human tears, which are responsible for repair mechanisms on the ocular surface^5–9^. These substitutes or adjuvants of tears can be utilized for the treatment of severe ocular surface pathologies due to their regenerative and healing properties. To date mainly autologous adult serum preparations have been used as tear adjuvants, but more recently neonatal derived products [cord blood (CB) and amniotic membrane (AM)] have also been investigated. Neonatal products have interesting characteristics including an excellent safety profile and unique composition due to the nature of donors. In fact, both amniotic membrane and cord blood serum have been used for the treatment of several conditions with varying degrees of success^7,9–13^. However, the mechanism of action is not well understood, and the variability in clinical outcomes poses the question as to whether this is due to variability in composition between different preparations and different donors, or if it may be influenced by the choice of preparation for each different indication.

The curative potential of human cord blood serum eye drops^13^ and human amniotic membrane extract eye drops^9,12^ has been recognized for some time, specifically their potential to decrease fibrosis and promote wound healing^11,13^ due to their cytokine and factor composition^13,14^. Less well characterised is CB platelet rich plasma, CBED’s starting material, which appears to be a more potent immune regulator than adult plasma^15^, in part due to presence of the soluble Natural Killer group 2D ligands (sNKG2DLs) such as MHC class I polypeptide-related sequence (MICA/B) and unique long 16 binding protein (ULBP1), which we have been previously shown to have an immunosuppressive effect on immune cells^16^. These sNKG2DLs are barely detectable in healthy individuals, but cell surface expression is induced when cells have been infected by viruses or become malignantly transformed. Their expression on the surface of “stressed” cells serves as a mechanism of natural killer (NK) cell activation and leads to the cytotoxic elimination of the infected/malignant cells and co-stimulation of CD8^+^ T-cells. During tumour progression, however, tumour cells can cleave these ligands shedding them as soluble forms which can bind the NKG2D receptor in NK and other cells thereby passively blocking it. Blocked NKG2D receptors bound to soluble ligands are internalised leading to receptor unavailability and downregulation of their activity, concomitant with impairment of the cell’s cytotoxic function^17^. This immunosuppressive mechanism is a classic example of tumour immune escape and it is thought to be used during pregnancy as a mechanism of fetal-maternal tolerance^18^. This mechanism, leading to immunosuppression, is unique to CB plasma as these molecules are not detectable in healthy adult plasma.

Previously, we validated manufacturing methods to produce neonatal tissue derived preparations: frozen CB platelet lysate^19^ and lyophilized AM extract, collected routinely from volunteer donations for either hematopoietic cell transplant or surgical procedures at a tissue bank, which are the precursor materials for the CBED and AMED preparations described here. The purpose of this study is to conduct a non-clinical assessment of these two novel allogeneic eye-drop preparations, used in a recent retrospective, case series clinical study^20^, which have not been characterised before, in order to facilitate the understanding of the mechanisms of action of the products in vivo, dosing and selection of preparation and/or donor according to indication. To characterise the preparations, we first measured their GF and cytokine profile and tested their regenerative potential and bioavailability in ex vivo model and we further tested their immune modulatory effects using a lymphocyte assay.

## Results

### CBED and AMED are rich in growth factors and cytokines

Results of GF and cytokine concentrations in both eye drops preparations are shown in Table 1 along-side those reported for tears in the literature as reference. The reported concentrations are of the diluted product as formulated for clinical use (see Materials and Methods). The reported concentrations are the mean of 10 independent batches (from 10 different donors) each measured in triplicate. The two preparations have different profiles. The concentrations of all factors measured was higher in CBED than AMED except that of basic fibroblast growth factor (bFGF). We observed variability in GF concentrations between samples (donors), which is reflected in the standard deviations (SDs) reported.

**Table 1:** GFs and cytokines concentrations in CBED and AMED.

Molecule	Tears^5,44^	CBED	AMED
**I. Trophic and wound healing factors**			
EGF	200–3000	254 ± 120	52 ± 21
bFGF		32 ± 14	112 ± 49
HGF	200–500	264 ± 98	151 ± 75
TGFβ1	2–10 × 10^3^	102 ± 25 × 10^3^	34 ± 8
**II. Angiogenic factors**			
VEGF	19	421 ± 240	13 ± 6
PDGFAB/BB	90–1700	3 ± 2 × 10^3^	1.9 ± 0.3
MMP-2	138–270 × 10^3^	150 ± 32 × 10^3^	400 ± 300
MMP-9	7–111 × 10^3^	87 ± 47 × 10^3^	102 ± 70
TIMP-1	66–93 × 10^3^	57 ± 11 × 10^3^	2 ± 1.5 × 10^3^
TIMP-2	71–183 × 10^3^	39 ± 3 × 10^3^	1.3 ± 1.2 × 10^3^
TIMP-3	1.5 × 10^3^	25 ± 8 × 10^3^	0.8 ± 0.1 × 10^3^
TIMP-4	1000	311 ± 150	Undetectable
**III. Pro-inflammatory cytokines**			
IL-1α		10 ± 5	6 ± 4
IL-6		9 ± 4	Undetectable
TNF-α		10 ± 2	Undetectable
**IV. Anti-inflammatory cytokines**			
IL-10		30 ± 15	Undetectable

Results are presented as mean and SD (n = 10) in pg/mL.

### Neither CBED or AMED were cytotoxic and both improved in vitro cell growth

**Figure 1:**
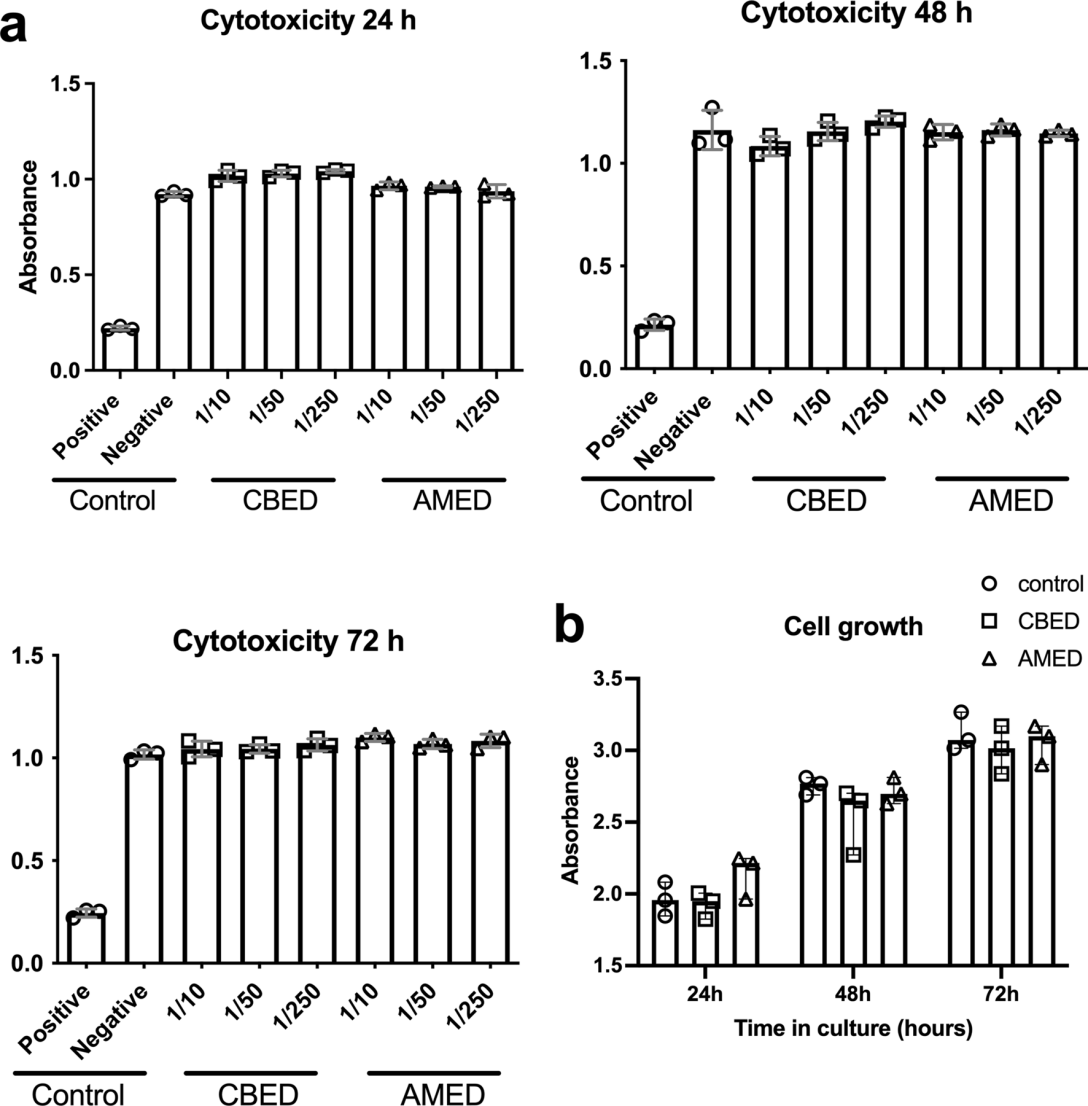
In vitro cytotoxicity and cellular growth assays performed with HCE cells. (**a**) Cytotoxicity was measured at 24, 48 and 72 h as a function of absorbance in a WST assay. Preparations were used at 1:10, 1:50 and 1:250 dilutions. SDS (0.02%) was used as a positive control (for cytotoxicity) and FBS (10%) as negative control. There are no statistically significant differences between any of the dilutions and the negative control (Mann–Whitney test used) (**b**) Cell growth was measured as a function of absorbance in a crystal violet elution assay. Absorbance is directly related to the cell density on the plates. Cells grown in either FBS (control), CBED or AMED have no statistically significant differences in cell densities after 24, 48 and 72 h of culture (2-way multiple comparison ANOVA with Tukey’s post-hoc test used).

Neither CBED nor AMED showed cytotoxic effects on HCE cells at any of the dilutions or time points (24–72 h) assessed using the WST1 cytotoxicity assay (Fig. 1a). The absorbance levels observed are comparable to those of the negative control (FBS) and well above those of the SDS treated cells (positive control). Using the colorimetric cellular mass approach crystal violet dye elution assay, we demonstrated that both CBED and AMED are capable of supporting cell growth**.** At 1/10 dilution in 5% FBS supplemented medium, both presented similar cell growth rates to those of control 10% FBS supplemented complete medium at all assayed time points (Fig. 1b).

### CBED and AMED support cell migration and close the scratched area in an in vitro wound healing assay

**Figure 2:**
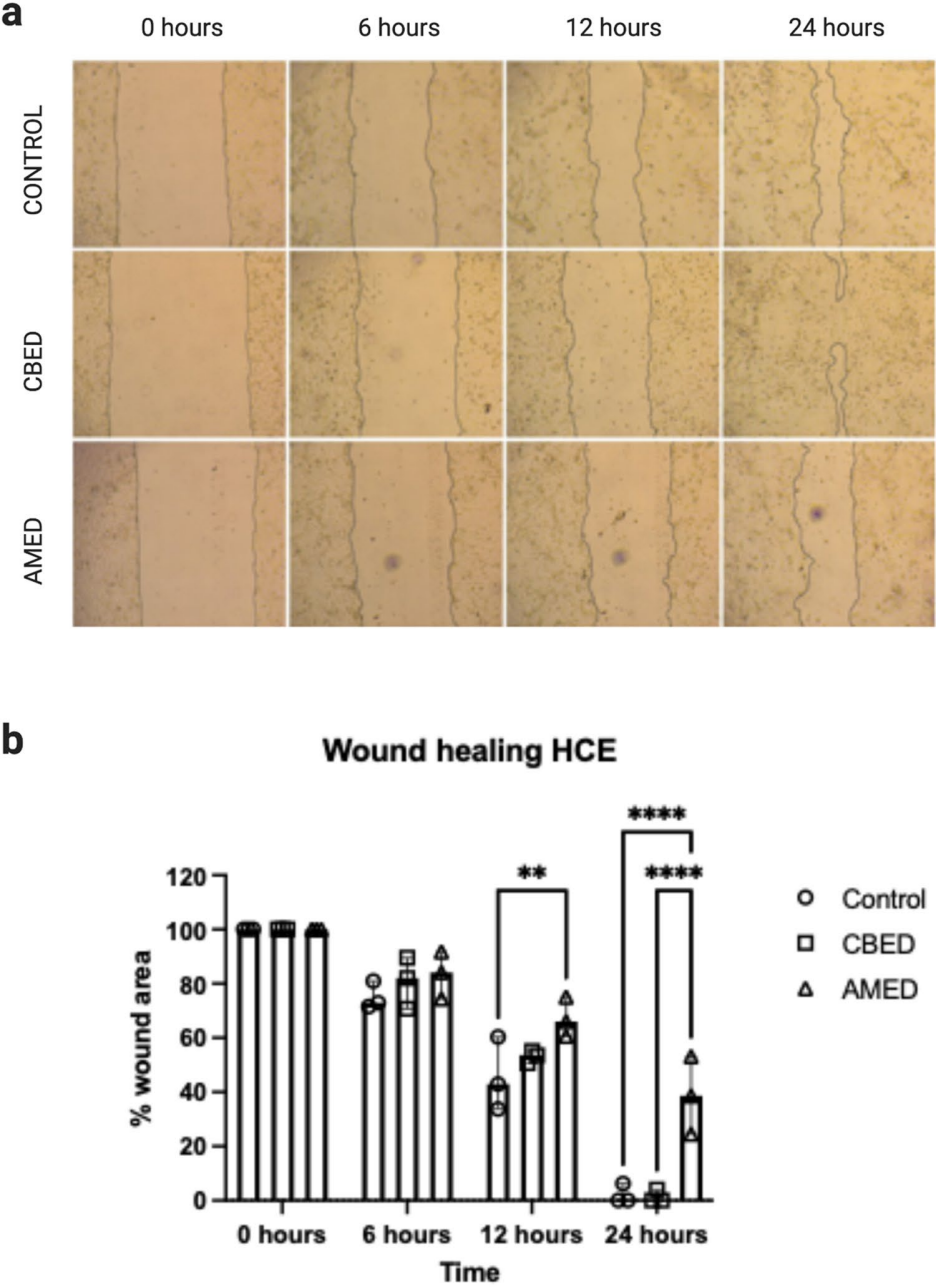
Scratch test wound healing assay using HCE cells. (**a**) Representative images of scratch assay using HCE cells. The scratched area was photographed and measured at baseline (0 h), 6, 12 and 24 h. Each product, CBED, AMED or control (FBS), was used at 10% concentration in complete medium. The images represent one of the replicates and area measured as wound. (**b**) Area closure in wound healing experiments (scratch test) was represented as percentage closure (%). Image J was used to calculate closure area. Bars show the median and range (n = 3). There are no statistically significant differences between CBED and control at any time point, however closure is significantly lower in AMED compared to control at 12- and 24-h time points (*p* = 0.0042 and *p* < 0.0001 respectively) and at 24 h also significantly lower than CBED (*p* < 0.0001) (2-way ANOVA with Tukey’s post-hoc test used) *p* < 0.01 (^**^); *p* < 0.001 (^***^), *p* < 0.0001 (^****^). (Figure created with BioRender.com).

To investigate the regenerative properties of the two preparations, we established an in vitro corneal “wound model” using HCE cells. A scratch (wound) was made on the HCE cell monolayer and its area was taken as baseline or 100% wound (3 wells were used per preparation). Cells cultured in media containing 10% foetal bovine serum (FBS) (positive control) and 1/10 (vol:vol) CBED or AMED migrated to close the area of the scratch. Within 12 h of culture around 50% of the scratch area had been closed by cell migration in all 3 culture conditions, with no significant differences between CBED and control or CBED and AMED, though in cultures treated with AMED the percentage closure was lower than control (*p* = 0.0042). By 24 h, however, while the scratch had been closed in 2/3 of the wells treated with FBS and CBED (6% and 4% of area remaining open in 1 well of each respectively), the area of the scratch remained larger (~ 38%) in the AMED treated wells (Fig. 2a, b).

### Growth factors present in the eye drop preparations are able to penetrate the corneal tissue

**Figure 3:**
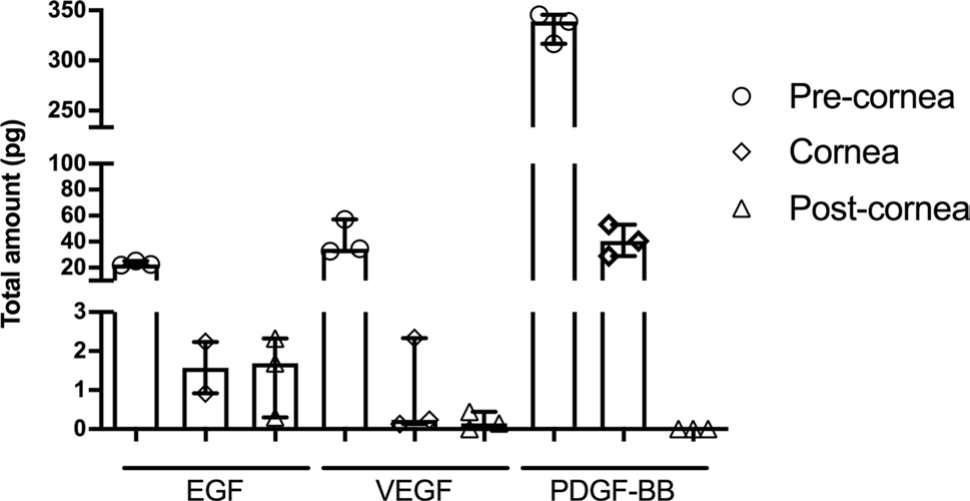
Bioavailability on different compartments on an ex vivo porcine corneal model. Results of ex-vivo permeation study showing the total amount (in pg) of three proteins: EGF, VEGF and PDGF-BB found in the 3 compartments: pre-corneal, corneal and post-corneal after application of CBED for 5 h in this model. Bars show the median and range.

Using the in vitro model of corneal penetration, we were able to measure the concentrations of some growth factors present in the two preparations (CBED and AMED) in the three compartments: pre-corneal, corneal and post-corneal. For all GF tested in CBED and AMED, most of the applied dose remained in the pre-cornea fraction. The concentration of GFs in AMED samples was below the detectable range in this assay. However, for CBED a variable but detectable fraction was found inside the corneal tissue. Concentrations (median and range) found were 1.6 (0.9–2.2), 0.2 (0.1–2.3), and 40.5(29–53) pg for epidermal growth factor (EGF), vascular endothelial growth factor (VEGF) and platelet derived growth factor (PDGF-BB), respectively. The concentrations at the pre-corneal compartment, were 22.6 (21.8–25.1), 34.7 (32.7–57.0) and 339.0 (316.7–345.7) pg, respectively. Finally, minimal amounts of EGF [1.7 pg (0.3–2.3)] and VEGF [0.15 pg (0–0.44)] were detected in the post-cornea fraction (Fig. 3).

### CBED and AMED have an immunosuppressive effect on activated lymphocytes

#### Both eye-drop preparations improve peripheral blood mononuclear cell (PBMC) viability

To ensure the preparations didn’t have an adverse effect of on the viability of immune cells, PBMCs were cultured in complete media or media containing 50% CBED or AMED. The percentage of live lymphocytes (7AAD- Annexin-) was determined after gating for CD3^−^ CD56^bright^/^dim^ NK cells, CD3^+^ CD56^+^ NKT cells and CD3^+^ CD56^−^ T cells (Figure S1, supplementary data). NK and NKT cells cultured in either CBED or AMED had higher viabilities compared to those in media alone, but the difference was only statistically significant for cells cultured in AMED. Both CD3^−^ CD56^dim^ and CD3^−^ CD56^bri^ NK cells showed improved viability when cultured with AMED samples compared with CBED (86.7 ± 2.3 and 92.2 ± 1.4% (*p* = 0.0042) compared to 95.2 ± 0.7 and 96.7 ± 1.5% (*p* = 0.0058) respectively). In CD3^+^ CD56^−^ T-cells there were no significant differences between cells cultured in media compared to the two preparations. However, when these cells were incubated with CBED, a mean viability of 93.2 ± 0.9% was achieved compared with 90.4 ± 1.0% when incubated with AMED, which was statistically significantly lower (*p* = 0.0031). Overall incubation of PBMCs with either CBED or AMED had no detrimental effects on the viability of the cells (Fig S2 supplementary data).

#### Incubation with CBED and AMED reduces expression of NKG2D

**Figure 4:**
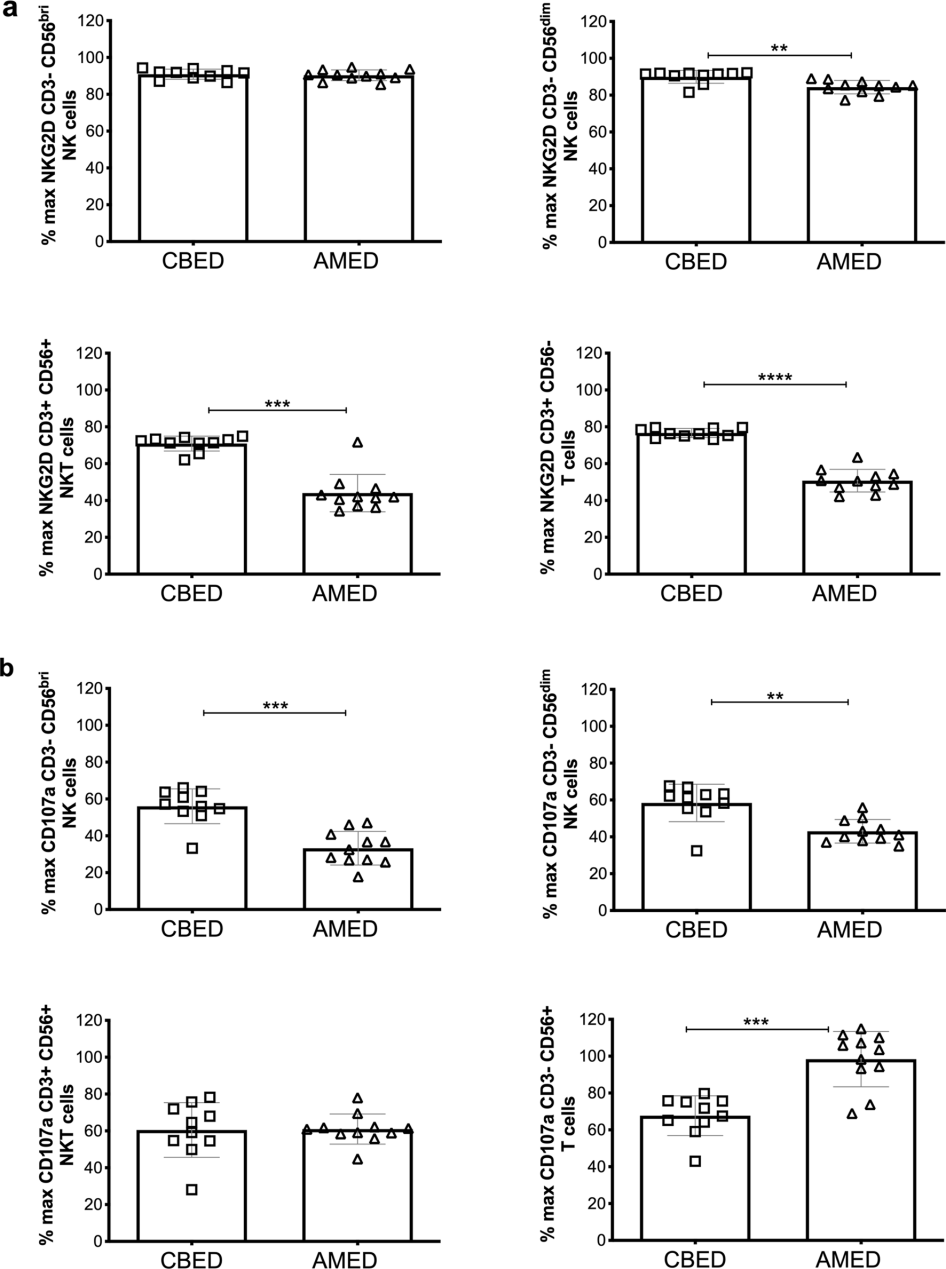
Expression of NKG2D and CD107a on NK, NKT and T cells from healthy donors is reduced by incubation with either CBED or AMED preparations. Results show percentage of maximum expression relative to complete media in CD3^−^ CD56^dim^, CD3^−^ CD56^bright^ NK cells, CD3^+^ CD56^+^ NKT cells and CD3^+^ CD56^-^ T cells from adult donor PBMCs after incubation with either eye drop preparation followed by PMA and ionomycin stimulation. (**a**) NKG2D (**b**) CD107a. Eye drop preparations investigated were CBED (n = 10) and AMED (n = 10). PBMCs were incubated with 50% eye drop solution (diluted with complete media and IL-2) or complete media and IL-2 only for 48 h prior to antibody staining and flow cytometry analysis. Each experiment was repeated with four different PBMC donors and data points represent donor means. Statistical analysis was performed using nonparametric Mann–Whitney test. *p* < 0.01 (^**^); *p* < 0.001 (^***^), *p* < 0.0001 (^****^).

Incubation of peripheral blood CD3^−^ CD56 dim and bright NK cells, CD3^+^ CD56^+^ NKT cells and CD3^+^ CD56^−^ T cells with either preparation resulted in a reduction in expression of NKG2D (Fig. 4a). AMED preparations reduced NKG2D expression more in all cell types analysed, except in CD3^−^ CD56^bri^ NK cells, where the reduction was not significantly different between the two. Incubation with AMED reduced NKG2D expression to 84.3 ± 3.6% of maximal in CD3^−^ CD56^dim^ NK cells compared to 89.9 ± 3.5% with CBED (*p* < 0.01), and to 50.7 ± 6.1% in CD3^+^ CD56^−^ T cells compared to 76.7 ± 2.4% with CBED (*p* < *0.0001*).

#### Incubation with CBED and AMED reduces expression of CD107a and IFN-γ

Expression of CD107a is associated with the process of degranulation and release of lytic granzymes^21^ and was reduced in most cell types tested upon incubation with AMED or CBED, with significant differences between the preparations and their effects on each cell type. Incubation with AMED reduced the expression of CD107a significantly more than CBED in both CD3^−^ CD56^dim^ (43.0 ± 6.3% compared to 58.4 ± 10.2%; *p* = 0.0021) and CD3^−^ CD56^bri^ NK cells (33.3 ± 9.1% compared to 56.0 ± 9.4%; *p* < 0.0001). This was, however, not the case in CD3^+^ CD56^−^ T cells, where there was hardly any reduction in expression upon cultivation with AMED (98.4 ± 15.0% of maximal), while CBED reduced it to 67.7 ± 10.8%; (*p* < 0.001) (Fig. 4b). This was unexpected as incubation with AMED had resulted in a more dramatic downregulation of NKG2D in these cells.

**Figure 5:**
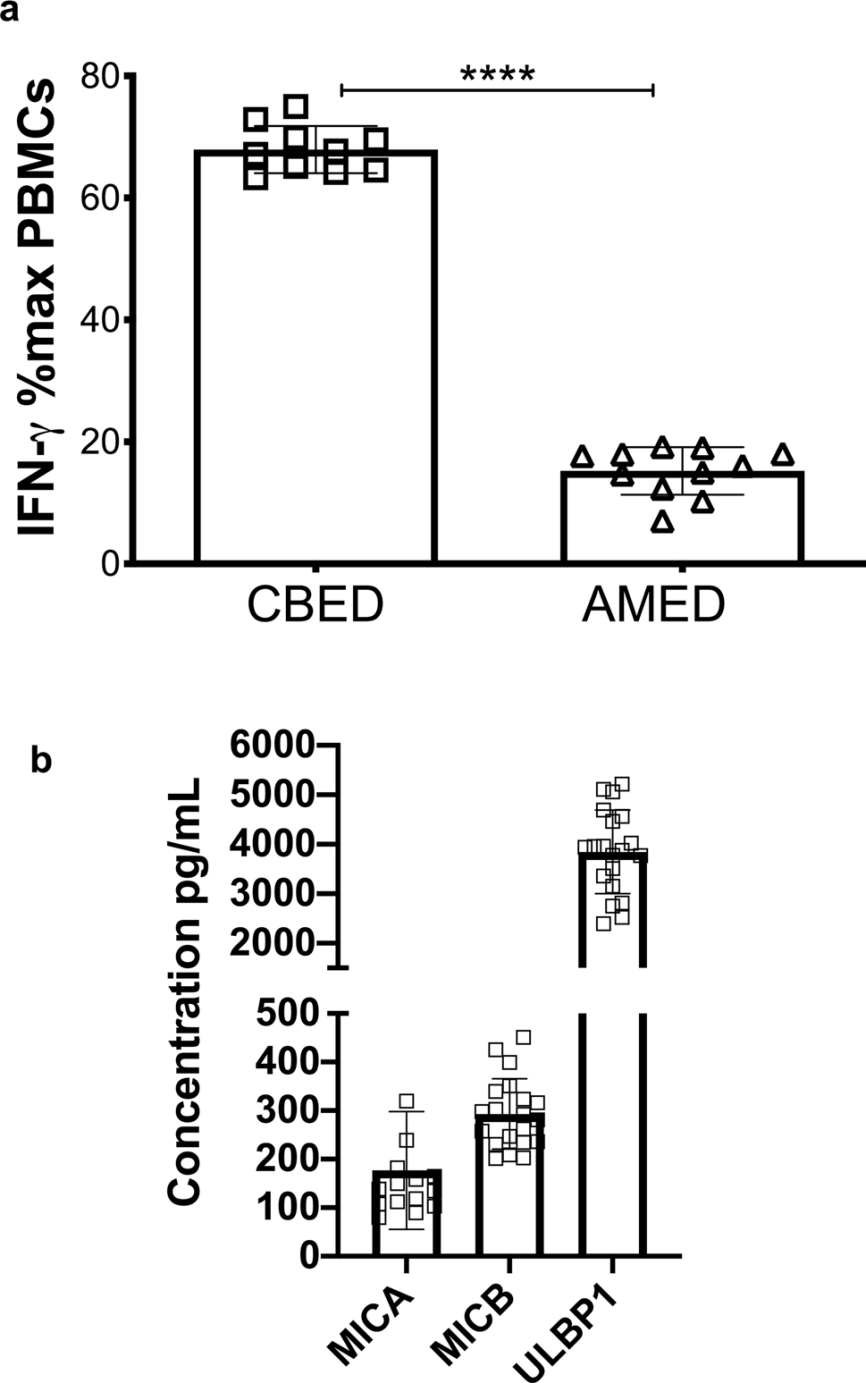
IFN-γ production by PBMCs from healthy donors is reduced by incubation with either CBED or AMED eye drop solutions. (**a**) Results show percentage of maximum IFN-γ expression in culture supernatants relative to complete media following 2-h stimulation with PMA and ionomycin. Eye drop preparations investigated were CBED (n = 10) and AMED (n = 10). PBMCs were cultured with 50% eye drop solution (diluted with complete media and IL-2) or complete media and IL-2 only, for 48 h prior to PMA and ionomycin stimulation. Each experiment was repeated with four different PBMC donors and data points represent donor means. (**b**) ELISA quantification of soluble NKG2D ligands (sMICA, sMICB and sULBP1) in CBED in pg/mL. Statistical analysis was performed using nonparametric Mann–Whitney test. *p* < 0.0001 (^****^).

We then quantified the total amount of IFN-γ produced after stimulation of cultures following the 2-h incubation period (Fig. 5a). Incubation with either eye-drop preparation resulted in reduced PBMC IFN-γ production compared to complete media only but to different extents. AMED reduced the expression much more dramatically than CBED, to 15.2 ± 3.9% of maximal compared to 67.9 ± 3.9% (*p* < 0.0001) when CBED was used.

#### CBED but not AMED contains sNKG2DLs

We previously found that CBP contains soluble NKG2DLs such a sMICA, sMICB and sULBP1^16,22^. Using ELISA, we measured the concentration of these ligands in CBED and AMED preparations. We found that sMICA ligands were detectable in 75% of CBED samples at concentrations of 176.8 ± 120 pg/mL and all samples had detectable levels of sMICB 293 ± 72 pg/mL and sULBP1 3846 ± 840 pg/mL, however no sNKG2DLs could be detected in any of the AMED samples (Fig. 5b).

## Discussion

In the present study, we describe the immunomodulatory and regenerative properties of CBED and AMED, two eye-drop preparations that have been recently used for the treatment of severe ocular surface diseases. We show that these placental derived preparations contain active molecules and GFs which are present in tears and are important for corneal healing. Both formulations promoted cell migration and growth with no cytotoxic effects on a human epithelial corneal cell line. Importantly, they suppress the activation of NK and CD8^+^ T cells in vitro and show corneal penetration in an ex-vivo model.

We observed a degree of variability (spread) in the concentrations of the different GFs between donors, which is expected from human derived products, and in line with previously reported GF concentrations measured in adult plasma^23^. The GF profile of each preparation may be used to establish optimal dosing and to best “match” individual preparations to patients according to the severity of the indication or other patient characteristics. The benefits of this protein profile is widely described in the literature; GFs such as PDGF, transforming growth factor beta (TGF-β), bFGF and VEGF have been shown to enhance regeneration in other tissues^24^. A topical supplement of EGF, usually found in tears, has the ability to induce and improve the process of corneal epithelial healing both in vitro and in vivo^1^. Together these findings suggest that topical application of those GFs would be beneficial as a treatment for corneal lesions, especially those requiring neovascularisation, such as chemical burns. From a practical point of view, CBED is rich in angiogenic factors, such as VEGF, which is 22-fold higher in CBED than in tears (see Table 1), that may improve chemotaxis of accessory molecules to promote ulcer closure in the first steps of stromal wound healing^1,25^ as well as TGF-β1 which is pivotal in ocular surface wound healing events^26^. It is important to note that the concentration of TGF-β is at least 5 times higher in plasma than in tears and that therefore in previous studies using autologous plasma for the treatment of dry-eye disease, the preparation was diluted to adjust the TGF-β concentration to those of tears^27^. However, because the pleiotropic effects of TGF-β may have differential effects depending on concentration and in the present study our primary objective is to find treatments for persistent eye injuries where there is a need to activate mechanisms of regeneration including angiogenesis, stem cell activation and some degree of inflammation^28^, we believe, the higher concentrations of TGF-β found in CBED would be beneficial. Equally the possibility that high concentrations of VEGF, specifically in CBED, could induce neovascularisation, which may compromise corneal transparency, cannot be ruled out. However, this has not been observed so far when autologous serum has been used in ophthalmic treatment, nevertheless it could be monitored closely during clinical application of CBED.

Similar to CBED, AM is a rich source of biologically active molecules and as such, promotes healing and acts as an effective material for wound closure, by supporting epithelialization and by displaying anti-fibrotic, anti-inflammatory, anti-angiogenic and antimicrobial features^29,30^. AMED contains many of the properties of cryopreserved or lyophilized AM. Among them, high levels of GFs^12^ including EGF, HGF, and bFGF, which under the topical formulation have the great advantage of providing a highly sustained concentration of trophic factors. Unlike CBED, AMED has low concentrations of VEGF, even lower than those in tears, which may make this preparation more desirable for the treatment of conditions where neovascularisation may be undesirable. In addition, in the ex vivo model of trans-corneal permeation used, we demonstrated the capacity of CBED to penetrate the corneal tissue where its effects are likely to be exerted. Importantly, we detected EGF, VEGF and PDGF-BB in corneal tissue, suggesting a direct influence in the process of corneal repair in vivo. Finally, the fact that negligible amount of GF appeared in the post-corneal chamber suggests a low risk of systemic effects.

The reduction in expression of NKG2D and CD107a seen in NK, NKT and CD8^+^ T cells incubated with either CBED or AMED is considerable, and it could explain one mechanism by which CB platelet rich plasma derived products (such as CBED) exert their immune-suppressive effects. We have previously shown that CB plasma contains soluble NKG2D ligands (sNKG2DLs) that are partly responsible for suppression of NK cell activity^22^. NKG2D is an activating receptor with a diverse repertoire of ligands including the highly polymorphic MICA and B and the ULBPs 1–6^31–34^. We showed that soluble ULBP1 (sULBP1) is the most abundant soluble ligand in CB plasma and it directly down-modulated NK cell cytotoxicity in a dose-dependent manner^16^. Here we confirm that at least CBED also contains high levels of the ligand ULBP1. Although TGF-β can also reduce surface expression of NKG2D^35^, our previous studies^16^ dispute this mechanism in CB plasma.

sNKG2DLs engage with NKG2D leading to internalization of the complex and thus reduce the availability of membrane bound receptor impairing the responsiveness of the NK/NKT and T cells^36,37^, including reduced IFN-γ production, decreased cytotoxicity and virtually no cell proliferation^16^. This mechanism of NK, NKT and T cell inactivation doesn’t, however, explain how incubation of cells with AMED downregulates the expression of both NKG2D and CD107a (at least in NK and NKT cells), as we could not detect sNKG2DLs in AMED. Therefore, an alternative mechanism must be at play, we suggest a likely combination of several other cytokines and factors present at high levels in AMED. It has been previously shown that human amnion derived cells are able to inhibit the function of NK cells by downregulating their activating receptors and reducing IFN-γ production^38^. Amniotic membrane cells are known to express HLA-G^39^, which is a ligand for NK cell inhibitory receptor KIR2DL4 and is thought to be involved in NK cell senescence during pregnancy to promote fetal-maternal tolerance^38^ in much the same way as sNKG2DLs. It is possible that AMED preparations contain soluble forms of HLA-G (5 and 6) that could explain why expression of CD107a and degranulation was inhibited in NK cells but not CD8^+^ T cells. Future studies are needed to investigate this.

Our results showed that the two preparations have different characteristics. For instance, CBED seems to be richer in growth factors both trophic and vascular, except bFGF that is higher in AMED. In contrast, immunosuppression seems more evident with AMED, but what is interesting is that the mechanism of action attributed as discussed above could be complementary: sNKG2DL *vs* HLA-G for CBED and AMED, respectively. Therefore, it is possible to envisage a combined therapy with both formulations. Characterising their composition and their regenerative and immunosuppressive effects will be helpful in understanding possible mechanisms of action and aid in formulation, dosing and choice of preparation for different disease indications.

An important aspect of our study is the fact that these products have been standardized in a tissue establishment and derived from routine donation in our CB and tissue banks. These type of preparations that are derived from donated birthing related tissues have some advantages over autologous approaches such are large-scale production of individual batches coming from a single donor, no donor age related variations, off-the-shelf availability, ready-to-use for patients in need. Lastly, as the product is prepared in advance, validation of product manufacture (under GMP), microbiological and safety testing as well as serological profiling can be carried out well in advance of product administration. These results are preliminary and need more testing, especially the influence of donor variability in their properties and the scalability for large production in a tissue establishment to support big clinical trials.

In conclusion, we have demonstrated that two routinely donated placenta derived tissues, can generate two different but complementary eye drops formulations, CBED and AMED, with regenerative and immune-suppressive properties that could be used to treat ocular surface disorders in which wound-healing is compromised.

## Materials and methods

### Study description and research ethics

This is an experimental study aiming to describe biological properties of human birthing related tissues (CB and AM) preparations currently used as eye drops in clinical trials. All research was performed in accordance with relevant guidelines and regulations of the Declaration of Helsinki and was approved by the local Research Ethics Committee of Hospital Clinic of Barcelona (HCB/2017/0785). All blood and tissue samples were obtained from volunteer donors within the authorized programs of the Banc de Sang i Teixits (BST) tissue establishment. Donation were obtained in a network of maternity services authorised for the activity of CB and placental collection. Informed consent was obtained from all participants that allowed sample transfer to BST’s research biobank which provided samples to the researchers. Experiments were performed at either BST’s Cell Therapy (Barcelona, Spain) or *Anthony Nolan Research Institute* (London, UK) research laboratories.

### Subjects and sample preparation

CB and AM donor eligibility for study enrolment was verified and samples taken tested negative for active transmissible infections, such as Hepatitis B and C, Human Immunodeficiency Virus, cytomegalovirus, *Treponema Pallidum* (Syphilis), HTLV I-II (human T-lymphotropic virus) and *Trypanosoma cruzi* (Chagas disease), using serological and NAT testing methods as well as microbiological analysis for aerobic, anaerobic bacteria and fungi according to local law requirements. Additionally, further processing of AMED and CBED was performed in clean room facilities at BST.

#### Preparation of CBED

Thirteen individual donor CB units (40% female, 60% males, from mothers ranging in age between 24.7 and 35.3 years and gestational weeks of 38.5 ± 1.2), which accomplished acceptance criteria were processed. Three were used exclusively for the corneal permeation assays, while the other 10 were used for all other experiments. Acceptance criteria included: < 48 h since collection, platelet content of > 150 × 10^9^ platelets/L, volume > 50 mL of cord blood (excluding anticoagulant citrate phosphate dextrose = 25 mL/bag) and absence of visible haemolysis.

To obtain standardized CB platelet concentrates (CBPC) the units were centrifuged twice (at 210 g and 2000 g in a Beckman Coulter Inc, USA, equipped with a SX4750A ARIES swinging bucket rotor). The acceptance criteria for CBPC^19,40^ includes: platelet count—1000 ± 200 × 10^9^/L, almost absence of leukocytes (≤ 0.5 × 10^9^/L) and erythrocytes (≤ 0.1 × 10^12^/L) in a volume 7–30 mL. These CBPC samples then underwent 3 freeze/thaw cycles^41^. After the third thaw cycle the CBPC was centrifuged at 5000*g* for 15 min to obtain the supernatant—a platelet lysate, rich in GF and cytokines, which was diluted with Plasmalyte (Baxter, Spain; 1:1, vol:vol) to obtain CBED^20^. Samples from each batch (donor) were aliquoted and stored at − 80 °C for further use.

#### Preparation of AMED

Thirteen batches (13 healthy donors) of standardized amniotic membrane extract AMED (maternal age 34 ± 3.4 years and gestational age 38.2 ± 1.3 weeks) were prepared from collected placentas and processed as follows^12,42^: the amniotic membrane was detached from the placenta and decontaminated in antibiotic solution RPMI 1640 (Corning, Manassas, USA), 10ug/mL amphotericin B (Xalabarder Pharmacy, Barcelona, Spain), 100 U/mL penicillin (ERN Laboratories, Barcelona, Spain), 2 mg/mL streptomycin (Normon Laboratories, Madrid, Spain). Next day the AM was washed in NaCl 0.9% to remove antibiotics, transferred to a Falcon tube (Thermo Fisher Scientific, Inc.) and flash frozen in liquid nitrogen immersion. The frozen AM was then ground (IKA, A11) and centrifuged at 4500 rpm at room temperature. The supernatant collected, was aliquoted in 1 mL fractions (vials), lyophilized under 2 days cycles (CoolVacuum, Barcelona, Spain), lyophilisation consisted of 3 phases: (1) phase 1—freezing of samples to − 40 °C at a pressure of 0.05 mBar, (2) phase 2—primary desiccation carried out at 10 °C and 2 mBar, and (3) phase 3—secondary desiccation carried out at 35 °C and 10 mBar, samples were then stored at room temperature until use. The lyophilized powder had approximately 145 ± 15 mg/mL. Experiments were performed with lyophilized AM extract, reconstituted in 4 mL sterile water (1:4, v/v), homogenized and then stored at − 80 °C until use.

The cellular assays were performed using peripheral blood mononuclear cells (PBMC) and human corneal epithelial cells (HCE). PBMCs were isolated by density-gradient centrifugation using Lympholyte-H solution (Cedarlane, ON, Canada) and subsequently used for the different cellular assays.

HCE were kindly provided by Dr E. Toropainen and Dr A. Urtti (University of Helsinki, Finland)^43^. HCE cells were cultured in complete medium consists of DMEM/F12 (1:1 v/v), EGF (10 ng/mL), insulin (5 µg/mL), DMSO (0.5%), Penicillin/Streptomycin (1%) (Sigma-Aldrich Inc, Darmstadt, Germany), and was supplemented with foetal bovine serum (FBS 5% or 10%) or experimental products (10%) in a humidified incubator at 37ºC with 5% CO_2_. Media were replaced every 48–72 h for cell expansion. HCE cells were used in cytotoxicity, cell growth and scratch wound healing assays as detailed below.

### Growth factor and cytokine assessment

CBED (derived from 10 CB units, 6 males and 4 females) and AMED (samples from 10 independent placental tissues) and prepared as detailed above, were further processed for analysis following manufacturer’s indications for each one of the GFs in the assay (Luminex Corporation USA), i.e. for TGF-β1 measurement, samples had to be activated by acid dilution before being measured. GF and cytokines were tested using the Luminex assays according to manufacturer’s instructions using xPONENT software, version 3.1 (Luminex Corporation, USA). The following analytes were assessed: trophic and wound healing factors (bFGF, EGF, HGF, TGFβ1); angiogenic factors (VEGF, PDGFAB/BB, MMP2, MMP9; TIMP1-4); pro-Inflammatory cytokines (IL-1α, IL-6, TNF-α), and anti-inflammatory cytokine (IL-10) (R&D Systems, Abingdon, UK). Three batches were selected to perform the next assays.

### Cytotoxicity assay

Functional cellular assays were carried out using standard established protocols for cell culture, previously described^43^. To ensure cell survival, the preparations tested had to be diluted in complete standard tissue culture media for testing at the dilutions described. In vitro cell toxicity was assessed using the WST-1 colorimetric assay (Cell Proliferation Reagent WST-1, Roche Applied Science, Mannheim, Germany) according to the manufacturer’s recommendations. HCEC were seeded at densities of 1–1.2 × 10^4^ cells/cm^2^ in 96 well plates. After 12 h of culture, various dilutions of CBED and AMED (1/10, 1/50 and 1/250) were applied in 5% FBS containing complete medium. After 24, 48 and 72 h of incubation, cell cultures were washed (× 5) with basal culture medium, and fresh complete medium containing 10% WST-1 reagent was added. After 3 h of incubation, absorbance was measured at 450 nm using an ELISA reader (Epoch; Bio-Tek Instruments Inc., Winooski, VT, USA) with corresponding software (Gen5, version 2.01). Control cell culture medium with 0.02% SDS (sodium dodecyl sulphate) was used as positive control for cytotoxicity. Complete culture medium with 10% FBS was used as a negative control. Three independent experiments (using preparations from 3 different donors) were carried out each one in triplicate (3 wells).

### Cellular growth assay

Cellular growth assays were conducted using crystal violet dye elution procedure as described previously^43^. In brief, HCEC were seeded at a density of 1–1.2 × 10^4^ cells/cm^2^ in 96 well plates, and after 24 h of growth, 1/10 dilution of assayed products (CBED and AMED) was added. After 24, 48 and 72 h of incubation, cell cultures were washed and incubated with a 0.25% solution of crystal violet in ethanol for 20 min. The dye was eluted for 30 min in 100 µL of 33% acetic acid. Absorbance was measured at 590 nm using an ELISA reader (DYNEX Technologies, VA, USA). Three independent experiments (using 3 different donors) were carried out in triplicate (3 wells).

### In vitro wound healing assay

HCEC were cultured in complete medium supplemented with 10% FBS for 48 h and then reduced to 2% FBS for 24 h. Cells were then seeded on 24-well plates and cultured until confluency. Scratching was carried out with a pipette tip, and images were taken under phase-contrast microscopy (Leica, Wetzlar, Germany) using a digital image acquisition program (LAS software, version 4.6). Cultures were treated with CBED and AMED at 1/10 dilution in complete medium without FBS. Controls were cultured in 10% FBS containing complete medium. Cultures were then photographed in the same location at 6, 12 and 24 h. The area of wound was measured using “Image J” software (https://imagej.nih.gov/ij/) and represented as the percentage of wound closure area by applying the following formula: 100 – [(wounded area at h/wounded area at 0 h) × 100]. For this experiment a representative sample of each preparation (CBED, AMED and FBS control) was used in three separate wells.

### Ex vivo corneal bioavailability assay

In order to assess transcorneal permeation the key GFs, EGF (for trophic), VEGF and PDGF-BB (for angiogenic) were selected for a bioavailability assay based on a porcine model using a Franz Chamber (SES GmbH—Analyse system, Model: V9CA-02) as described elsewhere^44^. Eyes were removed 4–5 h post-mortem from Landrace and Large White crossbred pigs (3–4 months old) slaughtered at the local abattoir (Barcelona-Spain). They were then kept refrigerated in buffered solution until use.

In brief, for this experiment three batches of samples were tested in 4 replicates of de-epithelized corneas. Three additional corneas were used as controls. Samples were applied in the donor chamber (pre-cornea). After 5 h of incubation, absorbed GF were recovered in the following compartments: donor chamber, inside corneal tissue and receptor chamber (post-cornea). Material obtained from the three compartments were lyophilized to concentrate the GFs and measure their level using a commercial immune-array following manufacturer’s instructions (QAH-ANG-1, RayBiotech, Peachtree Corners, GA, USA). Prior to their application in the array, the lyophilized pre-cornea and cornea fractions were resuspended in 300 µL miliQ water while the post-cornea sample was solubilized in 500 µL. Total concentration was calculated subtracting these values to the residual concentration in controls.

### In vitro immune modulation assay

For assessing immune effects of eye drops, PBMCs from 4 healthy adult donors were cultured (separately) in RPMI (Lonza, Slough, UK) supplemented with 1% penicillin and streptomycin, human IL-2 (1000 IU/mL) and either 10% heat-inactivated foetal calf serum (FCS) for the controls, or the same media diluted 1:1 with either CBED or AMED for the test cultures, in 96-well U-bottom plates in a humidified incubator at 37 °C with 5% CO_2_. After 48 h of culture, cells were treated with 100 ng/mL PMA, 1 µg ionomycin and 0.1% 2-mercaptoethanol (stimulated) or complete media with 0.1% 2-mercaptoethanol (non-stimulated) for 2 h at 37 °C. Following stimulation, expression of NKG2D, and CD107a was measured using flow cytometry. Unstimulated cells were used to measure base-line levels of expression of NGK2D, CD107a and INF-γ, and values subtracted from those in stimulated cells. Cells grown in control media were used as maximal expression and levels of expression in test cultures are expressed as % of maximal. Data points represent donor means of three replicate test samples.

The presence of INF-γ and soluble NKG2D ligands MICA/B (DY1300/DY1599) and ULBP1 (DY1380) was tested in CBED and AMED. IFN-γ (DY285) was detected in PBMC PMA and ionomycin stimulated supernatants. All assays were performed using Duoset ELISA kits (R&D Systems), according to manufacturer’s instructions.

Additionally, cells were analysed using flow cytometry. Briefly, cells were labelled with fluorochrome-conjugated antibodies in PBS with BSA (0.5%) for 10 min at 4 °C. To detect NK, NKT and T cells the following antibodies were used (BD Biosciences, Oxford, UK): CD3 (SK7), CD56 (B159), CD107a (HA4A3), NKG2D (BAT221, Miltenyi Biotec, Bisley, UK). Viability was assessed using Annexin-V and 7-AAD. Fluorescence minus-one (FMO) controls (where samples are stained sequentially with all antibodies except one) were used to set gates and analysis was performed using a Fortessa LSR flow cytometer (BD Biosciences, Oxford, UK) and FlowJo analysis software, version 10.0.8 (Tree Star Inc., OR, USA). The gating strategy used for analysis of lymphocyte subtypes was performed as described in our previously study^16^ (Figure S2, supplementary data).

### Statistical analysis

Statistical analysis was carried out according previous reports^19^. Results are shown as mean with standard deviation (SD) or median with ranges and were evaluated using Graphpad Prism 8 (Graphpad Software, CA, USA). Unpaired datasets were compared using the nonparametric Mann Whitney test. Where more than two groups were compared, analysis was performed using the Kruskal–Wallis test with Dunn’s post-hoc test. For multiple comparisons we used 2-way ANOVA with Tukey’s post-hoc test. A *p* value ≤ 0.05 in two-sided tests was considered significant. Significance levels are indicated as *p *≤ 0.05 (^*^), *p* < 0.01 (^**^), *p* < 0.001 (^***^) and *p* < 0.0001 (^****^), unless the exact *p* value is given.

## Supplementary Information

Supplementary Figure S1.

Supplementary Figure S2.

Supplementary Legends.
