# Supplementary figures and images for: Cord blood and amniotic membrane extract eye drop preparations display immune-suppressive and regenerative properties
Source: Sci Rep. 2021 Jul 2;11:13754. doi: 10.1038/s41598-021-93150-7 (PMC8253755; doi:10.1038/s41598-021-93150-7)

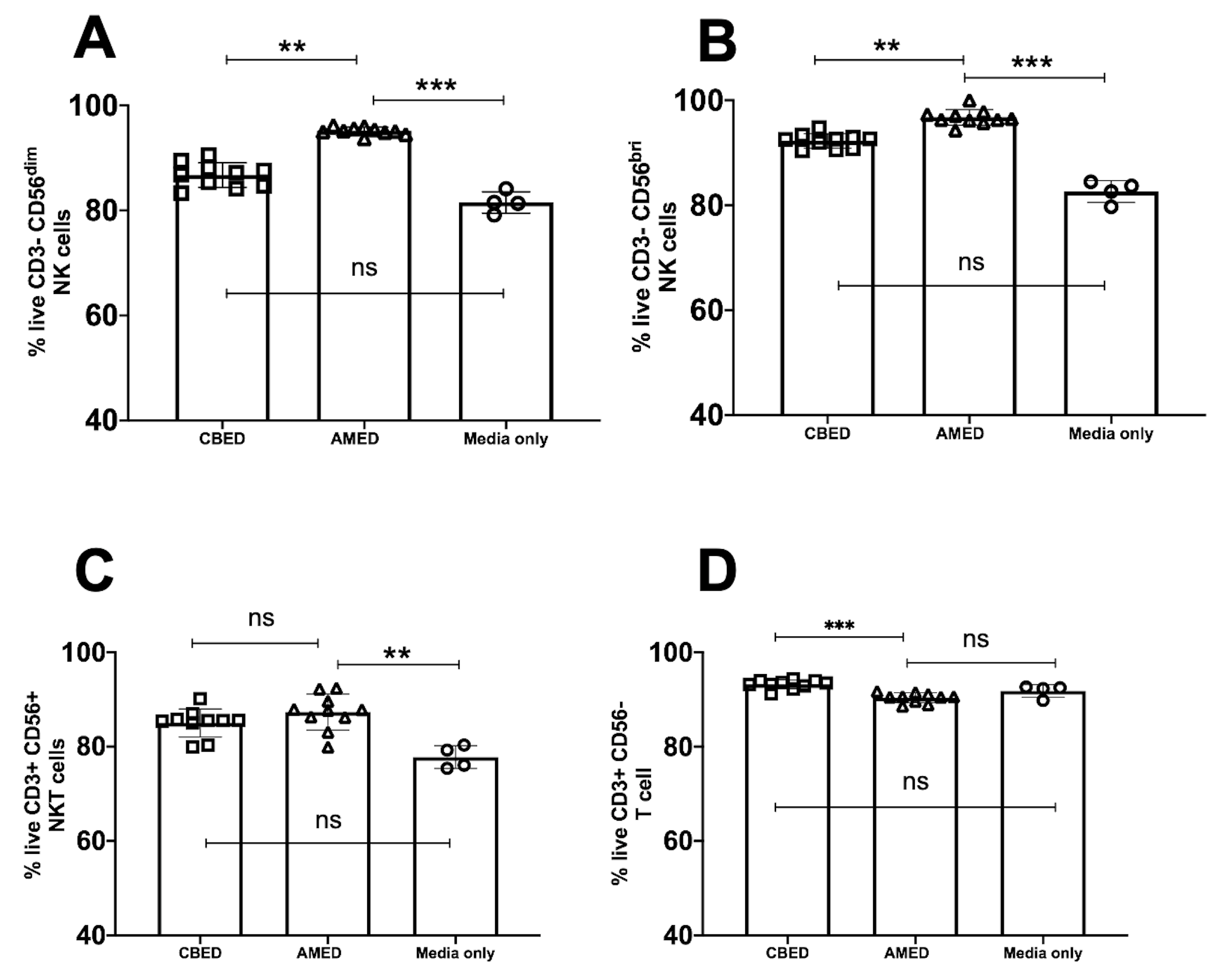

Supplement: Supplementary file 1 — Supplementary Figure S1. [file 41598_2021_93150_MOESM1_ESM.tiff]

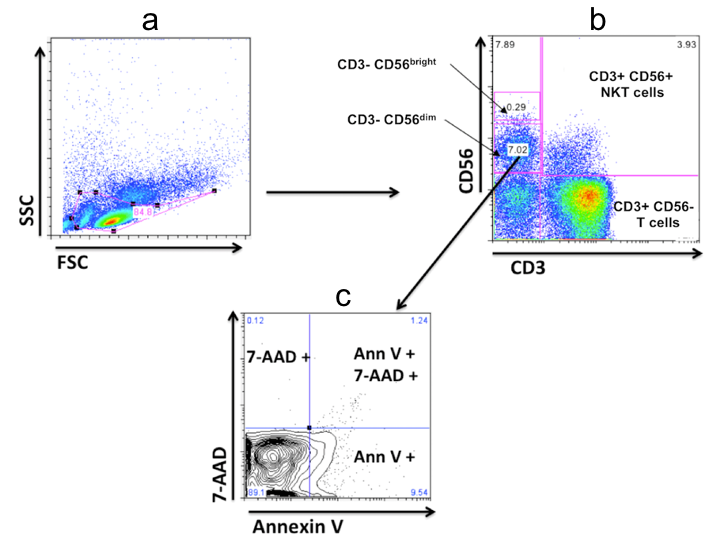

Supplement: Supplementary file 2 — Supplementary Figure S2. [file 41598_2021_93150_MOESM2_ESM.tiff]
