# Supplementary material for: Cord blood and amniotic membrane extract eye drop preparations display immune-suppressive and regenerative properties
Source: Sci Rep. 2021 Jul 2;11:13754. doi: 10.1038/s41598-021-93150-7 (PMC8253755; doi:10.1038/s41598-021-93150-7)
Supplement: Supplementary file 3 — Supplementary Legends. [file 41598_2021_93150_MOESM3_ESM.docx]

**Supplementary material**

*Ref: Submission ID 98431337-e51c-497a-98e9-88cce449a0e3*

*Title: “Cord blood and amniotic membrane extract eye drop preparations display immune-suppressive and regenerative properties” prepared by:*

*Dr. Dinara Samarkanova,*

*Dr. Steven Cox,*

*Dr. Diana Hernandez,*

*Dr. Luciano Rodriguez,*

*Dr. Maria Luisa Perez,*

*Prof. Alejandro Madrigal,*

*Ana Vilarrodona,*

*Dr. Sergio Querol,*

*Dr. Ricardo P Casaroli-Marano.*

**Figure S1. Viability analysis of CD3- CD56dim (A), CD3- CD56bright NK cells (B), CD3+ CD56+ NKT cells (C) and CD3+ CD56- T cells (D) in adult donor PBMCs.**Results show percentage of live cells (Annexin V negative, 7-AAD negative) for each cell type. Eye drop solutions investigated were CBED (n=10) and AMED (n=10). PBMCs were incubated with 50% eye drop solution (diluted with complete media and IL-2) or complete media and IL-2 only for 48 hours prior to antibody staining and flow cytometry analysis. Each experiment was repeated with four different PBMC donors and data points represent donor means. Statistical analysis was performed using nonparametric one-way ANOVA (Kruskal-Wallis test with Dunn’s post-hoc test for comparison with media only) or Mann-Whitney test (for comparisons between CBED and AMED). p<0.01 (**), p<0.001 (***), p< .0001 (****).

**Figure S2.  Gating strategy for determination of viable cells.** **(a)**  Forward and side scatter used for gating of lymphocytes populations. **(b).** From the lymphocyte population, NK, NKT and T cells were segregated according to the expression of CD3 (PE) and CD56 (APC) as shown. [NK cells- CD3-CD56^dim/bright^, NKT cells- CD3+CD56+, T cells-CD3+CD56-] **(c).** Each subpopulation in B was then analysed for cell viability using the 7-AAD fluorescent dye and annexin V (FITC). Viable cells are negative for both 7AAD and Annexin V. Gates were based on FMO controls (not shown).
